# Study of the Graphene Oxide Reduction Reaction Kinetics: Modeling and Electrical Monitoring of the Process

**DOI:** 10.1021/acsomega.6c06676

**Published:** 2026-10-05

**Authors:** Alessandra M. G. Barbosa, Andrés Vercik

**Affiliations:** † Departamento de Ciências Básicas, Faculdade de Zootecnia e Engenharia de Alimentos, 28133 Universidade de São Paulo, Av. Duque de Caxias Norte, 225, 13635-000 Pirassununga, SP, Brasil; ‡ Centro de Pesquisa Aplicada em Sustentabilidade Hídrica e Gestão de Recursos Ambientais (CEHIGERA), Faculdade de Zootecnia e Engenharia de Alimentos, Universidade de São Paulo, Av. Duque de Caxias Norte, 225, 13635-000 Pirassununga, SP, Brasil; § Bioengineering Graduate Program EESC/FMRP/IQSC-USP, Avenida Trabalhador São-Carlense, 400 - Parque Arnold Schimidt, 13566-590 São Carlos, SP, Brasil

## Abstract

Chemical reduction of graphene oxide is considered to be the most promising method for the large-scale, fast, and low-cost production of graphene in solution. Despite its importance, there is a lack of studies regarding this process, the detailed description of which would allow for optimization in terms of both time and cost. Moreover, a rapid and real-time method for monitoring the reaction could further optimize the procedure. The aim of this work is to study the kinetics of the chemical reduction of graphene oxide using ascorbic acid as a green reducing agent. For this purpose, the reduction reaction is monitored by measuring both the UV–vis spectra and the electrical conductance of the reaction solution, which increases as the reduction of graphene oxide proceeds. An accurate mathematical model of the reaction kinetics is proposed. Excellent agreement between the experimental and simulated curves was obtained using an uncoupled system of first-order differential equations for each reactant. The electrical monitoring of the reaction progress is validated by comparing the reduced graphene oxide concentrations obtained by the two methods, with no significant differences.

## Graphical abstract

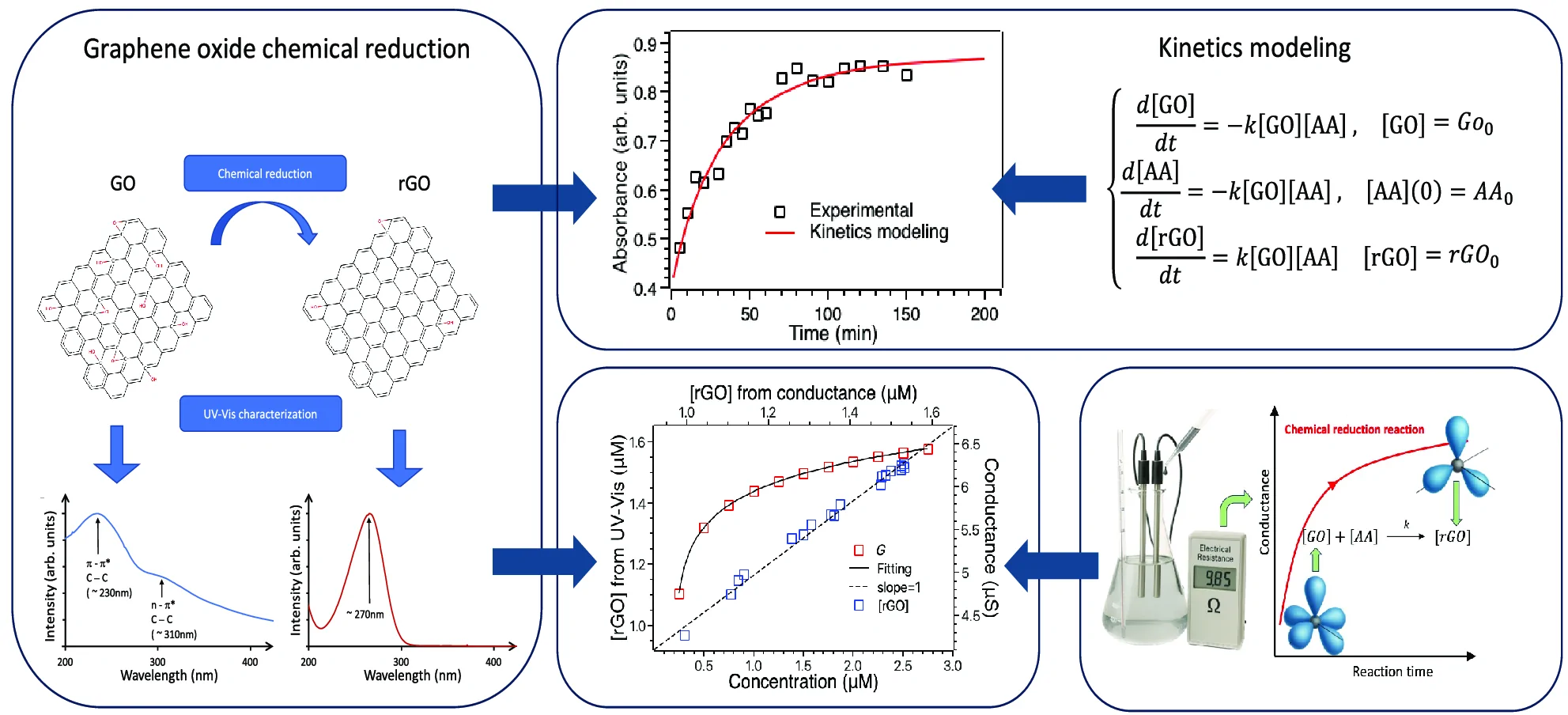

## Introduction

The discovery of graphene—the first two-dimensional (2D) material—by Geim and Novoselov in 2004 ^1–3^ revolutionized the field of nanomaterials. This was due to its astonishing properties, such as high electrical conductivity (10^5^ S/m),^4^ high thermal conductivity (exceeding 5000 W m^–1^ K^–1^),^5^ and high surface area of about 2640 m^2^/g (theoretical) and up to 750 m^2^/g (experimental).^6^ Consequently, graphene and graphene-related materials have a wide range of applications in electronics, energy conversion and storage, catalysis, sensors, and biomedicine, among others. Diverse methods exist to produce graphene, including mechanical exfoliation,^1,7^ epitaxial growth,^8^ chemical exfoliation of graphite,^9,10^ chemical vapor deposition,^11,12^ and the chemical reduction of graphene oxide (GO).^13–15^ Among these, the chemical reduction of GO is considered the most promising technique, as it potentially allows for the rapid, low-cost production of large quantities of graphene in solution.^16–19^ Furthermore, GO and reduced graphene oxide (rGO) find applications in areas where graphene obtained via other techniques is unsuitable, such as water desalination and purification.^20^ Nevertheless, a bottleneck persists in the large-scale production of high-quality, low-cost graphene, which currently limits the feasibility of some applications. Reports on graphite oxide date back to the 19th century.^21,22^ Its structure is similar to that of graphite, featuring oxygen-containing functional groups between carbon planes; it is referred to as graphene oxide when it consists of only a few layers. GO is a synthetic material whose production is extensively addressed in the literature.^23,24^ Currently, the most common procedure for obtaining GO is the method proposed by Hummers and Offeman in 1958,^25^ or similar eco-friendly modified techniques.^26^ The oxidation mechanism and chemical structure are usually described by the Lerf-Klinowski model,^27,28^ despite some controversy remains.^19^ According to this model, the carbon planes are decorated with hydroxyl and epoxy functional groups, while carbonyl groups are located at the edges.^29^ The high electrical conductivity of graphene, one of its most attractive properties, originates from the movement of π electrons along the conjugated structure. The functionalization present in GO disrupts this structure and localizes the electrons, diminishing carrier mobility. This leads to lower electrical conductivity due to the absence of percolating paths between sp^2^ islands. Consequently, GO is an insulating material, with a typical sheet resistance higher than 10 Ω/square.^30^ In order to recover a material that resembles graphene, with similar properties, GO can be subjected to a reduction process. GO reduction can be achieved through various methods, including thermal treatment,^31,32^ microwave photoreduction,^33–35^ and several modalities of chemical reduction.^14,23,36^ Chemical reduction is the most cost-effective method of reducing GO. A comparison and cost analysis of different chemical routes was published.^37^ Chemical reduction using hydrazine (N_2_H_4_)^38^ or sodium borohydride (NaBH_4_)^39^ is perhaps the most widely studied route, yielding rGO with excellent electrical and structural properties. However, the toxicity of these routes prevents the use of the obtained material in biological/environmental/food applications. Techniques utilizing nontoxic, nonpolluting methods and reactants, known as green routes, have gained significant attention recently,^16,19,40,41^ allowing, hence, environmental and biomedical applications, such as drug delivery and theranostics.^42,43^ Other green routes may involve biological reducing agents.^44,45^

The reduction of graphene oxide aims to remove oxygen-bearing functional groups, restoring the conjugated sp^2^ carbon framework and its associated π-electron system. These oxygen groups are geographically distinct: epoxide −O– and hydroxyl (−OH) groups primarily cover the basal plane, whereas carboxyl (−COOH) and carbonyl (=O) groups reside along the sheet edges. Because GO lacks a fixed stoichiometry, exact reaction pathways are strongly dependent on the chosen reducing agent and/or energy source. When using (N_2_H_4_), the reduction proceeds through a two-step mechanism. First, a nucleophilic amino group attacks an epoxide carbon on the basal plane, opening the ring to yield an amino-alcohol intermediate. This species then undergoes intramolecular substitution and elimination to form an aziridine-like structure, which decomposes into nitrogen gas and water to restore the C=C double bond:^38^

1 $$\mathrm{GO}+N_{2}H_{4}\to \mathrm{rGO}+N_{2}+H_{2}O$$

In the reduction with (NaBH_4_), the reducing agent acts as a H^–^ donor. The reduction occurs by hydride attack, converting carbonyl and epoxide groups into hydroxyl groups and subsequent dehydration that leads to partial sp^2^ conjugation restoration.

2 $$\mathrm{GO}+{{\mathrm{BH}}_{4}}^{-}+H_{2}O\to \mathrm{GO}+B(\mathrm{OH}{)}_{3}$$

NaBH_4_ is highly effective to reduce C=O and epoxide groups, but ineffective for reducing carboxylic acids or phenol-like hydroxyls.

Alternatively, l-ascorbic acid (C_6_H_8_O_6_) reduces epoxide and hydroxyl groups via proton and electron transfer. The enolate anion derived from ascorbic acid initiates a nucleophilic attack on the epoxide carbons. Subsequent hydrogen transfer and dehydration convert the oxygen moieties into water and regenerate C=C conjugation, while ascorbic acid oxidizes into dehydroascorbic acid (C_6_H_6_O_6_):^46^

3 $$\mathrm{GO}+C_{6}H_{8}O_{6}\to \mathrm{rGO}+C_{6}H_{6}O_{6}+H_{2}O$$

The characterization of both materials, GO and rGO, can be done by using different techniques, such as UV–visible (UV–vis) spectroscopy, X-ray diffractometry, Fourier transform infrared spectrophotometry (FTIR), X-ray photoelectron spectroscopy (XPS), and Raman spectroscopy.^19,29,47,48^ Among these techniques, UV–vis is less expensive and time-consuming, allowing fast sampling in order to study the evolution of the reduction reaction, which is one of the aims of this work. This can be done because GO and rGO have distinct characteristic spectra, which makes it possible to track them during the reaction. The unreduced GO has two well-defined features observed in the UV–vis spectrum: a primary peak maximum (λ_max_) located at approximately 230 nm due to π → π* transitions and a shoulder at around 300 nm due to electronic transitions of nonbonding lone pair electrons (*n*) present on oxygen atoms in carbonyl (C=O) and carboxyl (COOH) groups promoting to antibonding π* orbitals. These features are shown in Figure 1, where the characteristic structures of GO and rGO are shown, together with their respective UV–vis spectra. The structural changes produced by the removal of oxygen functional groups and the restoration of the conjugated carbon lattice during the chemical reduction are, in general, associated with two different modifications of the UV–vis absorption spectra: (1) a redshift of the primary π → π* peak from 230 nm toward 260–270 nm, and (2) an attenuation of the shoulder. These changes are explained structurally by the rehybridization from the distorted sp^3^ configuration characteristic of GO to a planar sp^2^ configuration typical of graphene, in the case of the main peak, and a breaking of the carbonyl-containing functional groups, eliminating the *n* → π* transition as the density of nonbonding oxygen pairs vanishes. As discussed later, we propose a different interpretation of these changes.

**Figure 1:**
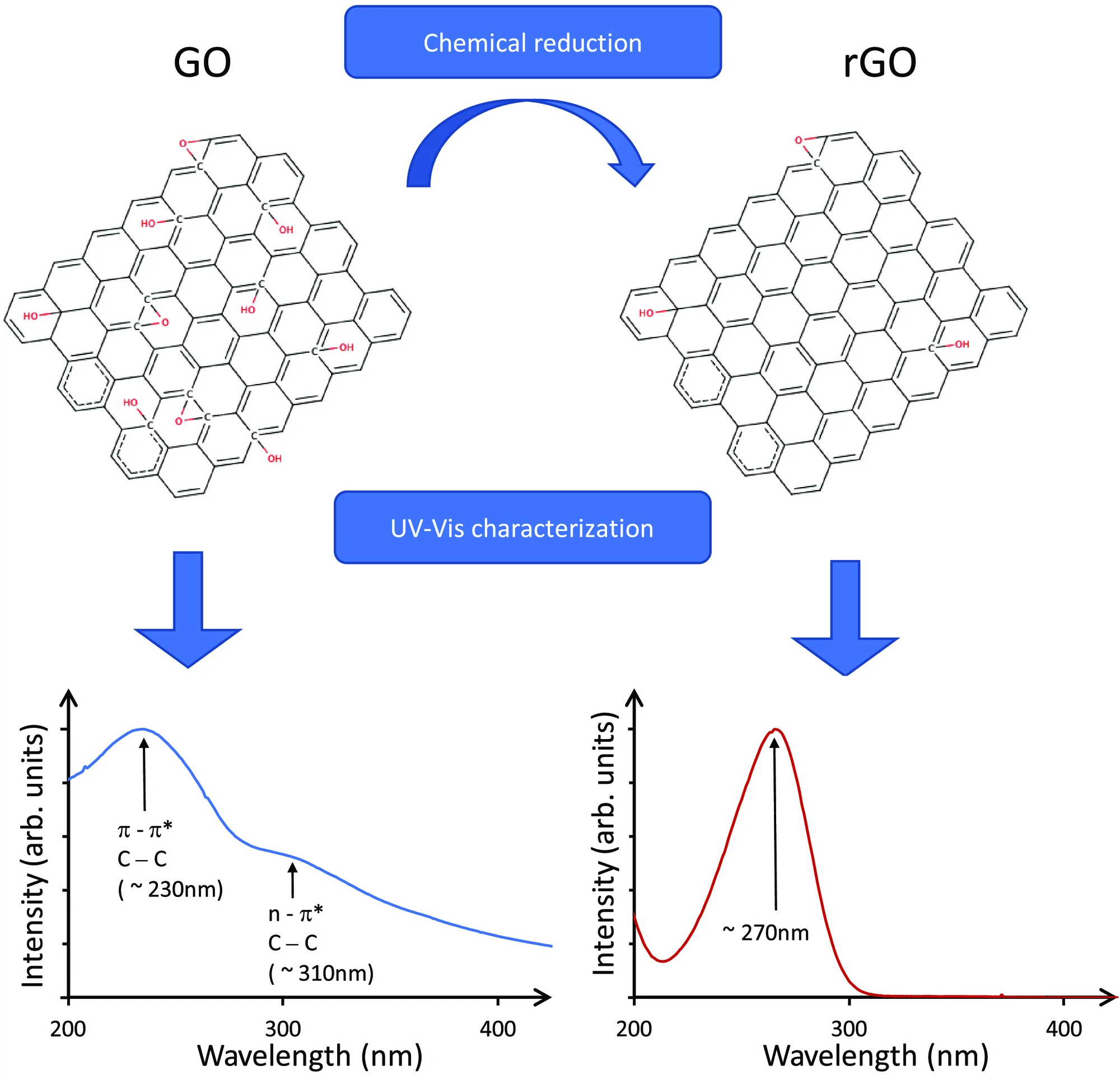
Chemical structure of GO and rGO. Each material has a characteristic UV–vis spectrum with different fingerprints: (i) a peak at 230 nm and a shoulder at 310 nm for GO and (ii) a unique peak at 270 nm for rGO. These features can be used to monitor the presence of each component in a reaction solution.

The kinetics of the reduction process is scarcely addressed in the literature. Zhang et al.^18^ addressed the time evolution of the reduction products by UV–vis, FTIR, and Raman techniques and other techniques, such as AFM and XPS, to analyze starting and final materials. They mentioned an observed redshift. However, the time intervals for measurements are 12 to 24 h, much longer than the reduction time. Therefore, other effects might be present. De Silva et al.^47^ studied the progress of the reduction reaction by using UV–vis and XRD, with 10 min sampling. They observed the above-mentioned redshift in the UV–vis spectra. Another important observation is that the XRD exhibits both peaks of GO and rGO for the first 30 min of reaction until the disappearance of the GO peak. A discussion later on shows that this observation, which is confirmed by other reports in the literature, is not compatible with the redshift interpretation. Wang et al. have made a detailed study of the green reduction using tea extracts^49^ and also have pointed out the redshift, which again is a result of a misinterpretation of the spectra produced by the superposition of different characteristics (GO, rGO, and background). Beyond that, there is also a lack of work modeling the kinetics, which can confirm the assumptions made. The aim of this work is to bridge this gap in studies regarding such an important subject, as the reduction of graphene oxide, taking into account the many scientific, technological, and commercial applications of this material.

Despite the simplicity of using UV–vis to monitor reaction kinetics, the necessity of manual sampling imposes a limit on temporal resolution. While this is not critical for a process lasting several hours, it would be advantageous to measure the evolution of the reaction in real time. In this work, a study of the reduction reaction via a nontoxic and eco-friendly route, using ascorbic acid as a reducing agent, is presented. The reduction reaction is monitored by UV–vis spectroscopy and also by measuring the electrical conductance of the reaction solution using a system of cylindrical electrodes for that purpose. As the reaction proceeds, as shown in Figure 2, the solution must become more conductive with the presence of rGO instead of the insulating GO, accompanying the change from sp^3^ to sp^2^ hybridization. Furthermore, a mathematical model for the reduction reaction is proposed on the basis of a simple system of first-order ordinary differential equations. Predicting the degree of GO reduction through modeling could enable the production of rGO with controlled electrical properties tailored to specific applications, leading to more efficient and cost-effective processing. The possibility of monitoring the degree and evolution of the reduction process was proposed and discussed.

**Figure 2:**
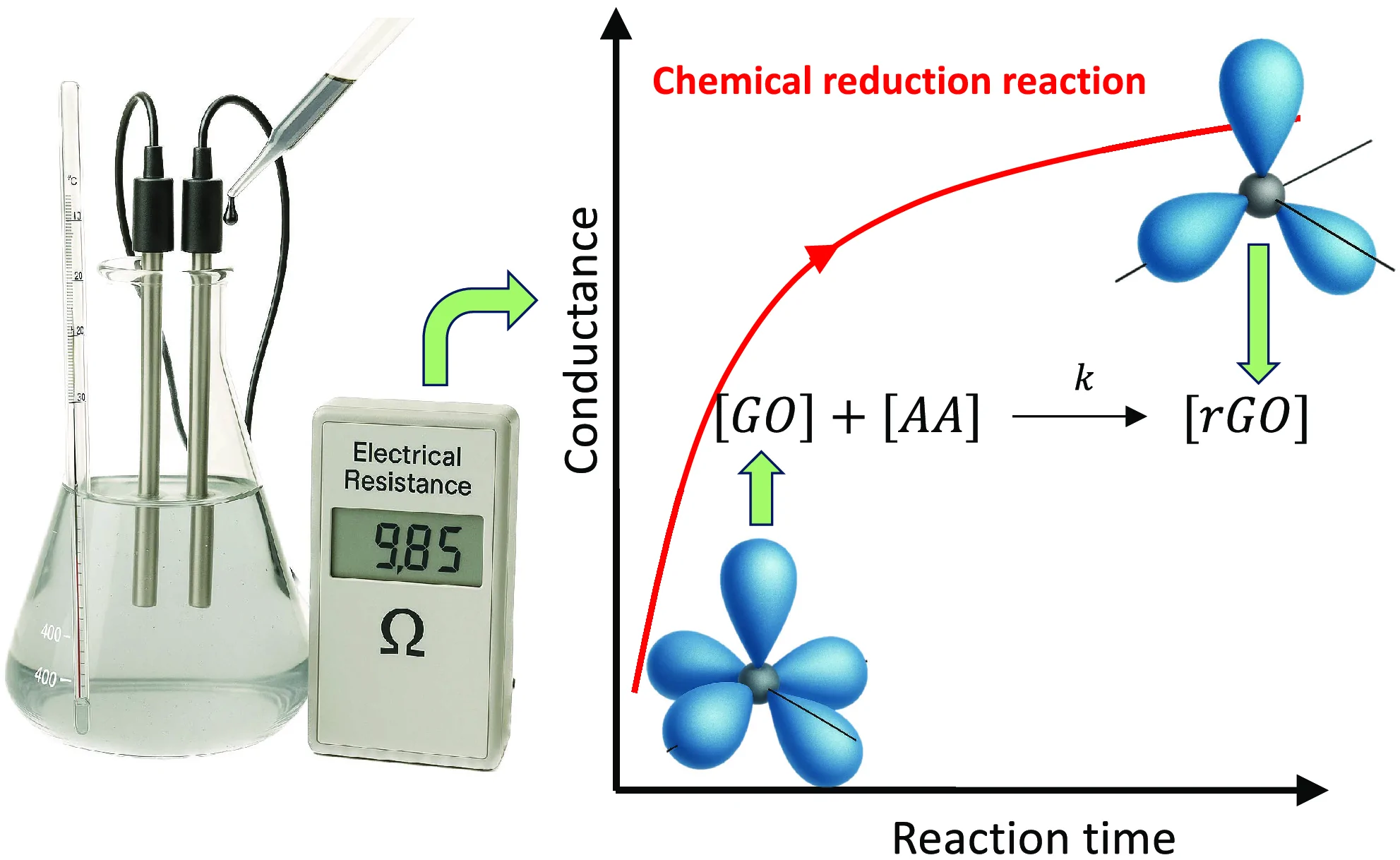
Schematic representation of the electrical monitoring of the reduction process. Two cylindrical electrodes were placed in the solution and connected to a multimeter for measuring the electrical conductivity. As reduction proceeds, the sp^2^ molecular orbitals are recovered and the solution becomes more conductive. Source: own authorship.

## Experimental Methods

### Graphene Oxide Synthesis

The synthesis of graphene oxide was accomplished by the modified Hummers method.^25^ For that purpose, 3 g of pure graphite powder was mixed with 70 mL of concentrated sulfuric acid (H_2_SO_4_) under constant stirring in a 1 L beaker flask immersed in ice (exothermic reaction). Subsequently, 9 g of potassium permanganate was added, and after stirring for 5 min, the solution was placed in a bath at 40 °C for 30 min. Then, 150 mL of deionized water (Milli-Q) was added to the solution, which was left in a bath at 90 °C for another 15 min. After this process, 500 mL of deionized water was added together with 15 mL of 30% hydrogen peroxide. At this point, a yellowish solution was observed, turning into a brownish solution. After the reaction ended, the solution was filtered under vacuum with a 14 μm filter paper, and finally, 250 mL of 10% hydrochloric acid was added to remove the metallic graphite ions. The resulting material was characterized by X-ray, UV–vis, and FTIR spectroscopies to verify the successful production of GO.

### Graphene Oxide Reduction

The reduction of GO was accomplished by using ascorbic acid (AA). The reduction started from a 0.1 mg/mL solution of graphene oxide, and then 0.1 g of AA was added under stirring, adjusting the pH of the solution with ammonia to values between 9 and 10 for colloidal stabilization of the GO suspension. The solution was sonicated for 30 min and then heated at 95 °C under stirring for approximately 2 h.

### Characterization

#### X-ray Diffraction and Fourier Transform Infrared Spectroscopy

The starting material (GO) and the final product of the reaction (rGO) were characterized by X-ray diffraction (XRD) and Fourier transform infrared (FTIR) spectroscopy. XRD patterns of GO and rGO samples were recorded over a 2θ range of 5–60 °, using an X-ray diffractometer (Rigaku, model Miniflex600, Japan), with a step angle of 2°/min. The FTIR spectra of the GO and rGO samples were measured using an FTIR spectrophotometer (PerkinElmer Spectrum One, Germany) in the wavenumber range of 400–3500 cm ^–1^ with a resolution of 4 cm ^–1^, in absorbance mode.

#### UV/Vis Spectroscopy

During the reduction process, sampling of the solution was used to measure the UV–vis absorbance with a Bell M51 spectrophotometer. The first measurement was performed after less than 1 min of the reaction. During the first hour, the time intervals for measurement were 5 min before the first hour, 15 min after the first hour, and 30 min during the second hour. No further changes in the solution were observed after 100 min, so the reaction was considered complete as the absorbance reached a stationary value. Calibration curves for GO, AA, and rGO were measured to quantify the concentrations of reactants and product.

#### Electrical Monitoring

The reduction was also monitored by placing two cylindrical electrodes into the flask, where the reduction was taking place. The conductivity of the solution was measured with a Fluke 8846A 6–1/2 digit precision multimeter.

## Results and Discussion

Figure 3 exhibits the preliminary characterization by (a) XRD and (b) FTIR spectroscopy of unreacted GO and the final rGO, after the reaction is complete. The reaction is supposed to be complete once a stationary UV–vis intensity value is achieved, which occurred after approximately 150 min of reaction, as determined in preliminary tests. The XRD spectra show the typical peak at 10.7° for the GO, with a replica at 27°. In the case of rGO, a much weaker peak is observed at 22.6°. These features are in good agreement with the literature^48,50,51^ and correspond to an interlamellar spacing of 0.83 nm for GO and 0.39 nm for rGO, which also agree with the values reported in the literature.^50^ The FTIR spectrum for GO displays a series of absorption bands corresponding to various oxygen functional groups introduced during oxidation:^50,51^ a broad band between 3200 and 3500 cm ^–1^ corresponding to O–H stretching vibration from hydroxyl groups and intercalated/adsorbed water molecules, a peak at around ∼1720–1735 cm^–1^ due to C = O stretching from carbonyl and carboxyl groups (predominantly located at the sheet edges), a peak at ∼1620 cm^–1^ due to C=C stretching corresponding to unoxidized graphitic sp^2^ domains, which appears to be overlapped with a band that can be associated with O–H bending mode of absorbed water, a broad band at ∼1360–1400 cm^–1^ due to O–H deformation and/or C–OH stretching vibrations, which can screen an unresolved band due to C–O–C stretching characteristic of epoxy/epoxide groups on the basal plane expected at ∼1220–1250 cm^–1^ and finally a peak at ∼1050–1080 cm^–1^ due to C–O stretching of alkoxy and hydroxyl groups. In the case of rGO, after the reduction, oxygen-bearing functional groups are removed, leading to the suppression or attenuation of most of the bands associated with oxygen-containing groups, remaining a weaker peak at ∼1600 cm^–1^ due to the C=C stretching band.

**Figure 3:**
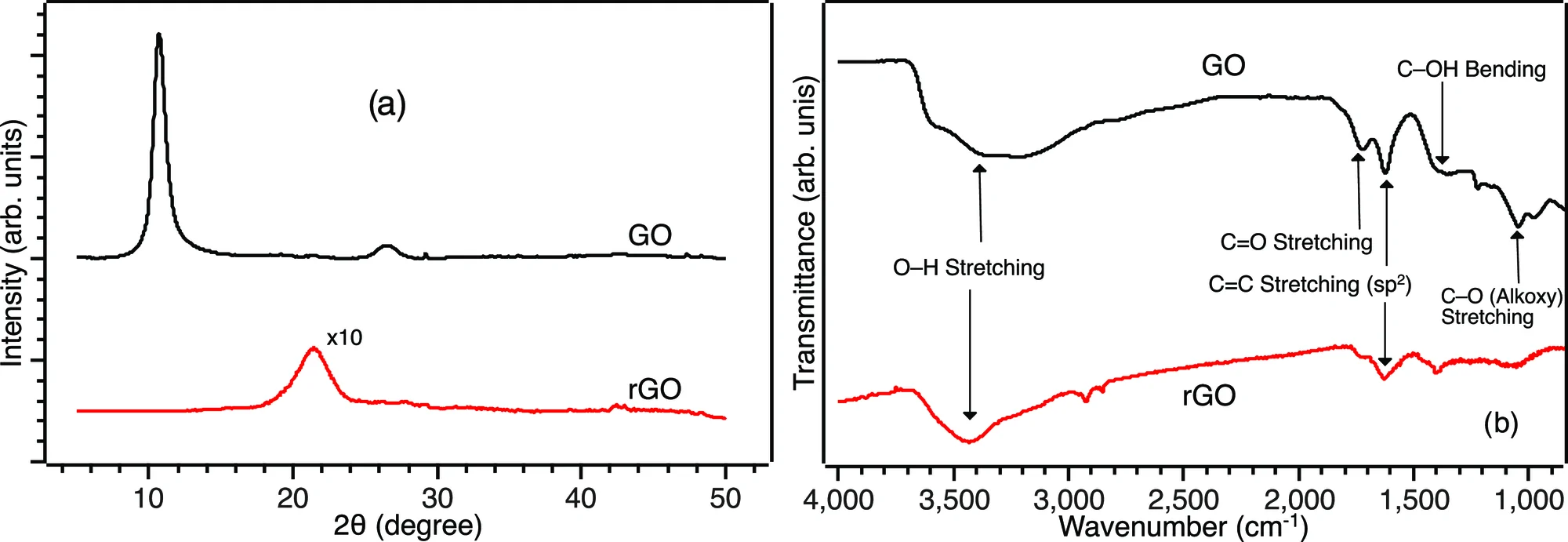
Characterization of the starting material (GO) and the product of the reduction reaction (rGO): (a) X-ray diffraction spectra and (b) FTIR spectra. These results are in good agreement with reports in the literature. Although these techniques confirm the results obtained by UV–vis spectroscopy, they are not suitable for real-time monitoring of the kinetics due to the time and resource consumption.

Although XRD and FTIR techniques serve to guarantee that the materials (starting and product) are the expected ones and that the reduction process has taken place, these techniques are not suitable for real-time (or at least fast sampling) monitoring of the reaction kinetics due to time and resource consumption and, for the purpose of monitoring the progress of the reaction, a better option is using UV–vis, following methodologies reported in the literature earlier^4,47^ . The UV–visible spectra of the reactants (GO and AA) and the product (rGO) were measured before starting the reduction procedures, as shown in Figure 4a, where the normalized spectra are plotted to identify the wavelengths of the characteristic features. As expected, the typical features, mentioned above, for GO and rGO are clearly observed. Thus, these measurements can be used to monitor the evolution of the reaction. Figure 4b shows several spectra measured throughout the procedure (reaction times indicated in the figure). The AA and rGO peaks cannot be rigorously resolved. However, since a low AA concentration was used, its peak was considered to be masked by the rGO peak. Furthermore, the maximum absorbance, which is proportional to the reactant concentration, is much stronger for rGO than for GO and AA. Calibration curves for each reactant are shown in Figure 4c, where linear fittings of GO, AA (both 100 times augmented), and rGO data are plotted. The three curves exhibit a highly linear relation with *R* ^2^ values very close to unity. As shown in Figure 4b, the reduction reaction occurs almost immediately after mixing the reactants. Figure 4d shows a comparison of the UV–vis spectra shapes of unreacted GO and of the solution at three different stages of the reduction reaction. Particularly, the deconvolution of the first two spectra in that figure is shown in Figure 4e,f, respectively, in order to identify precisely the coexistence of GO and rGO. As mentioned, the unreacted GO spectrum Figure 4e is composed of two Gaussian peaks at 236 and 314 nm, as expected. The deconvolution of the spectrum at the initial stage of the reaction Figure 4f suggests that three contributions are present: two of them at λ_max_ of 234 and 316 nm associated with the peak and shoulder typical of GO, and a third Gaussian peak at λ_max_= 265 nm corresponding to the rGO signal. In later stages, the product peak becomes stronger and dominant, and any trace of GO disappears. These curves clearly suggest a completely different interpretation of the spectra when compared with those works found in the literature.^18,47,49^ Although those authors mention a redshift of the peak from 230 nm toward 270 nm, the results presented here indicate a disappearance of the GO peak and enhancement of the rGO peak, which is more compatible with the consumption of one of the reactants (GO) and a production of rGO during the reaction. This misinterpretation of the spectral shift could be related to artifacts originating from the overlapping of peaks with an initial decaying background typical of UV–vis spectra or even between peaks of GO and rGO. Our interpretation of these results is more coherent not only with a scenario of consumption of reagents and emergence of the product, as mentioned, but also agrees more closely with the simulations presented later. Moreover, the peak shift picture seems not compatible with the simultaneous coexistence of both materials, GO and rGO, during the reaction reported in the literature. Effectively, AFM results^46,49^ indicate that both materials are present during the reaction, in good agreement with the deconvolution of peaks shown in Figure 4f, observable at the early stages of the reduction reaction.

**Figure 4:**
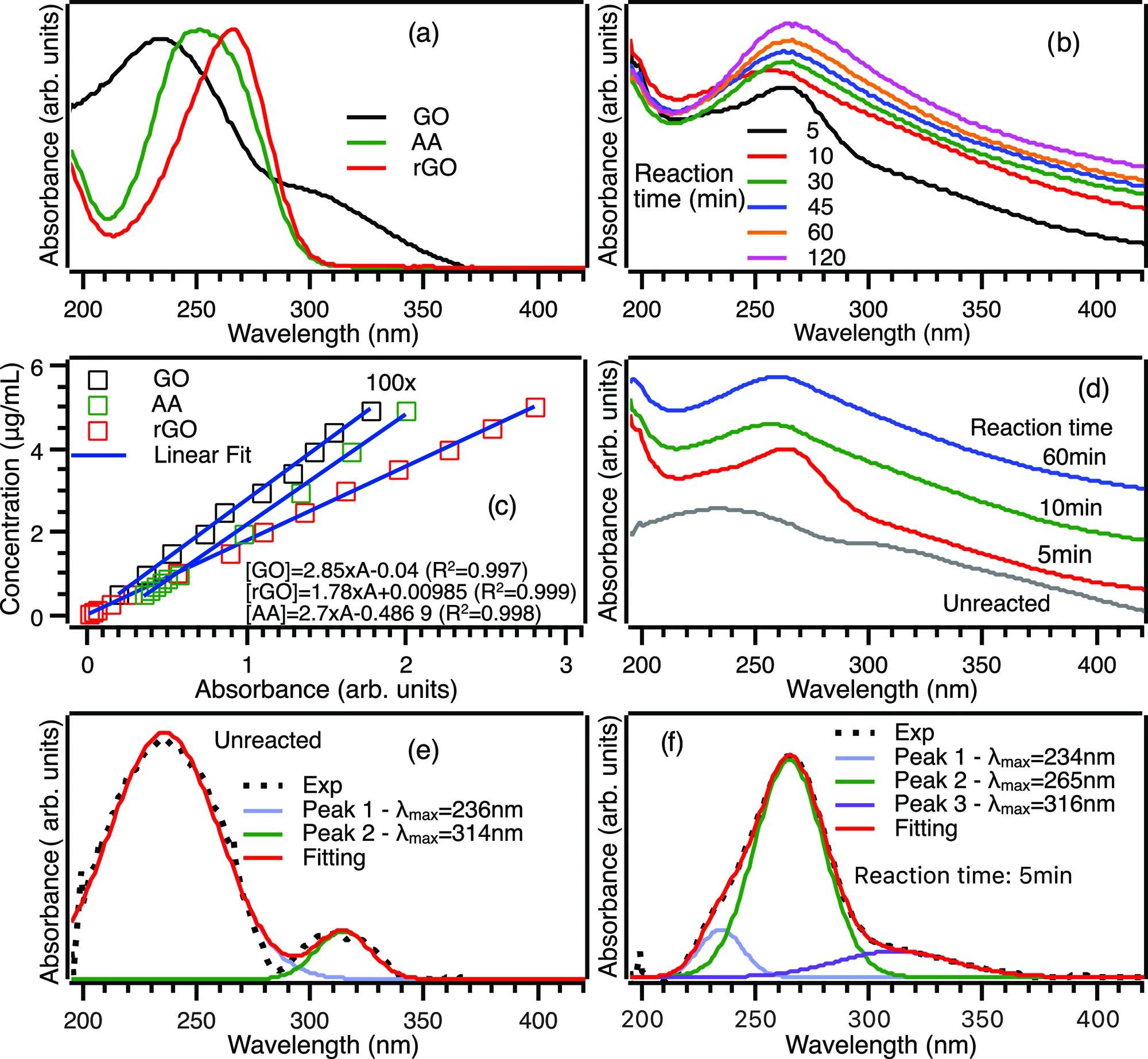
(a) Normalized UV–vis spectra of the reactants and the product involved in the reduction reaction: graphene oxide (black), ascorbic acid (green) and reduced graphene oxide (red); (b) UV–vis spectra measured during the reduction process (reaction times indicated in the figure); (c) calibration curves for reactants (GO and AA) and product (rGO); (d) details of the spectral shapes for unreacted GO and rGO at initial and final stages. The change from two GO peaks to three GO/rGO and then one rGO peak is clear. Deconvolution of (e) GO and (f) initial rGO spectra showing the presence of different contributions.

The reaction kinetics can be modeled according to simple principles:^52^

1. A nonstoichiometric reaction of reactants with concentrations [GO], [AA], and product concentration [rGO] was considered according to
4 $$\mathrm{GO}+\mathrm{AA}\overset{k}{\to }\mathrm{rGO}$$
2. The reaction occurs when both reactants are present; thus, consumption and production rates are proportional to [GO]·[AA] (first-order reaction);
3. The reaction stops when one of the reactants is exhausted.

The resulting system of equations is the following:

5 $$\{\begin{array}{l} \frac{d\left[GO\right]}{dt}=-k\left[GO\right]\left[AA\right]\\ \frac{d\left[AA\right]}{dt}=-k\left[GO\right]\left[AA\right]\\ \frac{d\left[rGO\right]}{dt}=k\left[GO\right]\left[AA\right] \end{array}$$

where [GO](0) = GO_0_, [AA] (0) = AA_0_, [rGO](0) = rGO_0_ are the initial values for the time-dependent concentrations [GO]­(*t*), [AA]­(*t*) and [rGO]­(*t*), respectively, with GO_0_> AA_0_ and *k* is the reaction rate. The solutions of the system in eq 5 are

6a $$\left[\mathrm{GO}\right]\left(t\right)=\frac{GO_{0}\left(GO_{0}-AA_{0}\right)}{GO_{0}-AA_{0}e^{-k\left(GO_{0}-AA_{0}\right)t}}$$

6b $$\left[\mathrm{AA}\right]\left(t\right)=\frac{AA_{0}\left(GO_{0}-AA_{0}\right)e^{-k\left(GO_{0}-AA_{0}\right)t}}{GO_{0}-AA_{0}e^{-k\left(GO_{0}-AA_{0}\right)t}}$$

6c $$\left[\mathrm{rGO}\right]\left(t\right)=rGO_{0}+\frac{GO_{0}AA_{0}\left(1-e^{-k\left(GO_{0}-AA_{0}\right)t}\right)}{GO_{0}-AA_{0}e^{-k\left(GO_{0}-AA_{0}\right)t}}$$

The solutions of the system of equations (eqs 6–8) are shown in Figure 5a. It can be seen that the product [rGO] increases from the initial value, whereas reactants [GO] and [AA] diminish. The stationary value of [rGO] is achieved once one of the reactants ([AA] in this case) is completely consumed ([AA] = 0). Since a nonstoichiometric reaction is considered, residuals of the other reactant ([GO]) are expected. The experimental data were fitted with this model, as shown in Figure 5b. The values of the fitting parameters (GO_0_, AA_0_, *k*, and rGO_0_) are presented in Table 1. Ideally, a zero initial value for rGO would be expected. However, as mentioned before, the reaction seems to initiate immediately after the reactants are mixed, so a nonzero initial condition has been considered. The experimental data correspond to the maximum absorbance peak extracted from deconvolution of the UV–vis spectra at different times (square symbols). An excellent agreement between experimental data and the model is observed, indicating the correct interpretation of the reaction. Figure 5b (triangles) also shows the values extracted from the work by Wang et al.^49^ A similar model can be used to fit those experimental results as well, as shown in the figure, with parameter values also presented in Table 1.

**Figure 5:**
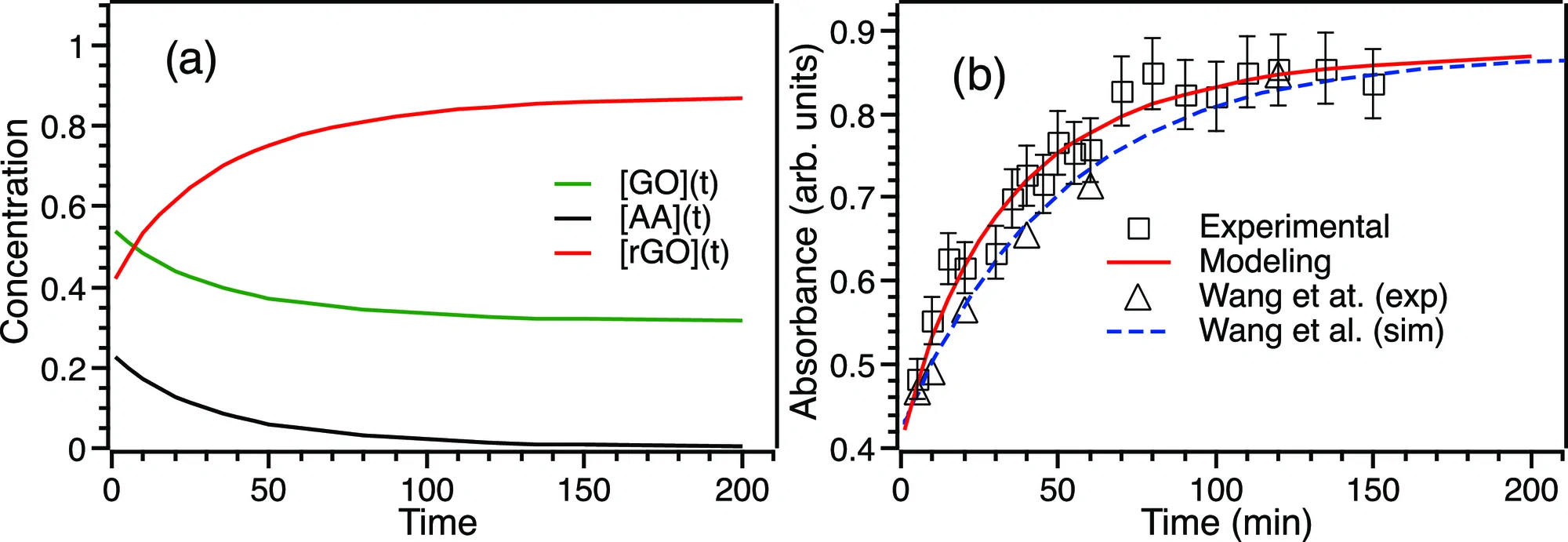
(a) Solutions of the system of equations (eqs 6–8) for the reactants [GO] and [AA] and the product of the reaction [rGO]. (b) Experimental intensities (squares) extracted from the rGO spectra during the reaction and mathematical modeling (red solid line). The experimental data (triangles) by Wang et al.^15^ and the corresponding modeling with eqs 6–8 are also shown.

**Table 1:** Fitting Parameters Used for Kinetics Modeling of the Product rGO during GO Reduction with Ascorbic Acid (AA)^a^

parameter	this work	Wang et al.^49^
GO_0_	0.55 ± 0.07	61 ± 5
AA_0_	0.23 ± 0.03	0.23 ± 0.01
*k*	0.06 ± 0.02	0.0003 ± 0.0002
rGO_0_	0.40 ± 0.03	0.02 ± 0.01
χ^2^	0.0103	0.00088
*R* ^2^	0.999	0.999

a The parameters for fitting the experiment by Wang et al. (extracted from ref 49) are also shown.

Finally, the reaction can also be monitored by measuring the electrical conductivity of the solution. For that purpose, a system with two stainless steel cylindrical electrodes was designed. Before using it in the reaction solution, a calibration process was performed, in order to take into account material and geometry factors of the conductometric setup. For that purpose, the electrical resistance (*R*) was continuously measured while a solution with known and controlled concentration of rGO was poured into a 0.1 mg/mL solution of GO (considering that in the reduction setup residuals of GO could be present in the reaction solution). The measured resistance and its inverse, the conductance (*G*), are plotted versus the rGO concentration in Figure 6a. Each step in *R* (black curve) or *G* (red curve) corresponds to the addition of rGO into the solution. The final conductance points, i.e., obtained from the *G*–*t* curve after stabilization for each drop added versus the concentrations of rGO, are plotted in Figure 2b (red squares). The concentration and steady conductance values are presented in Table 2.

**Figure 6:**
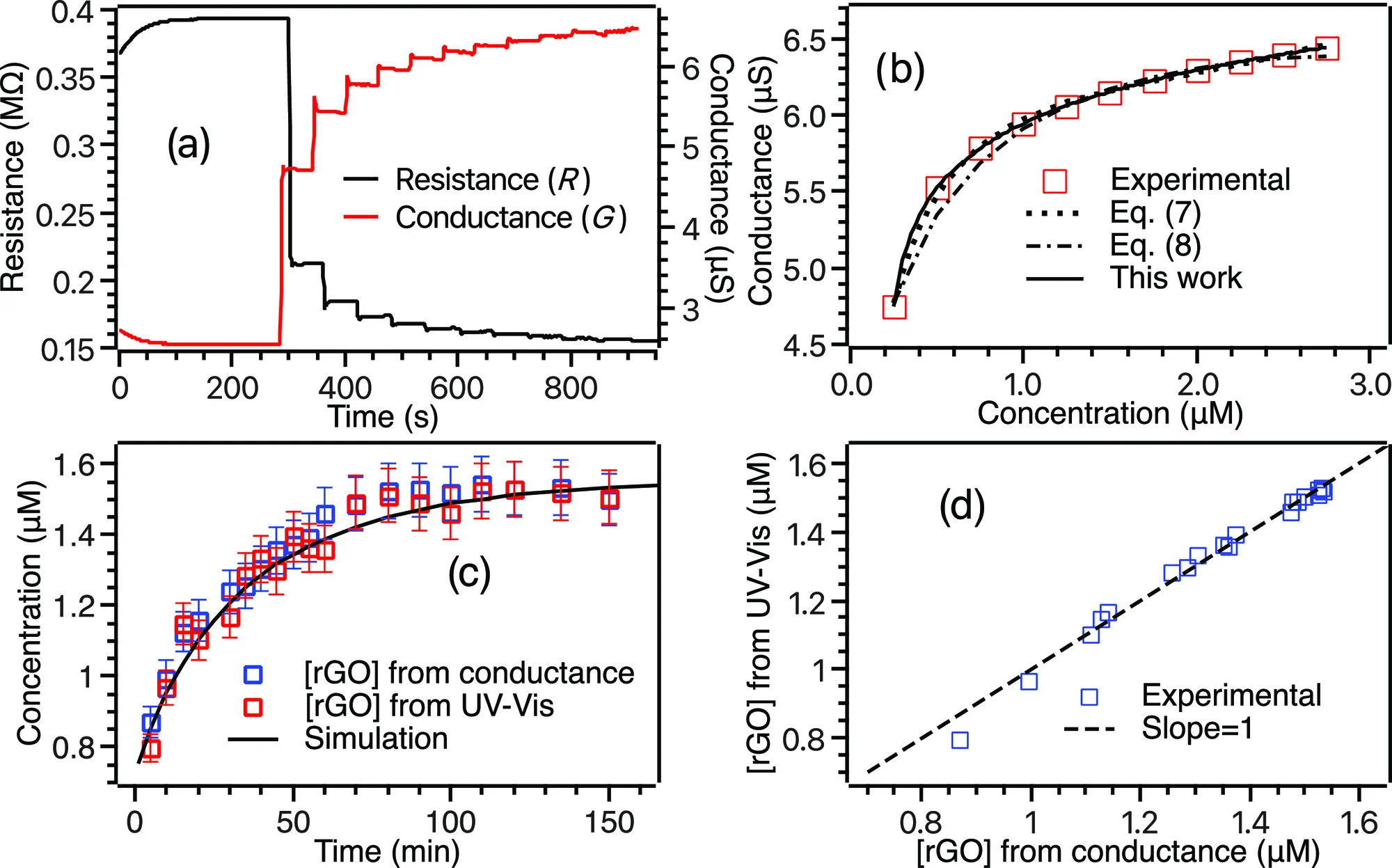
Electrical monitoring of the reduced graphene oxide concentration: (a) values of the resistance, *R*, measured during calibration of the technique (black curve) and its inverse, conductance *G* (red curve). (b) The valleys in the conductance curve are plotted versus the concentration of rGO (red squares). Three fitting models of the conductance–concentration curves were tested: known models—eq 9 (dotted line), eq 10 (dash-dot line), and the proposed eq 11 (solid line), with advantages for the expression proposed in this work. (c) Comparison of the rGO concentrations computed from conductance measurements (blue symbols) and from UV–vis spectra (red symbols) with the simulation using the proposed model (eqs 6–8). (d) Comparison between values of [rGO] obtained from the electrical measurement and the optical technique (blue squares). A straight line with unit slope is also shown.

**Table 2:** Concentrations Used for the Calibration of Conductance Measurements

concentration (μM)	conductance (μS)
0.25	4.752
0.5	5.522
0.75	5.783
1	5.944
1.25	6.061
1.5	6.153
1.75	6.228
2	6.293
2.25	6.349
2.5	6.398
2.75	6.442

For the proposed technique to be useful, an interpolation function relating rGO concentration and conductance is needed. Different expressions have been explored to take into account this dependence, such as the Fuoss–Onsager equation for the conductance–concentration relationship:^53^

7 $$\Lambda =\Lambda_{0}-S\sqrt{c}+Ec\;\log c+Jc$$

or the model for the conductivity of electrolyte solutions:^54^

8 $$\kappa =A\cdot m^{n}\cdot \exp\left(-B\cdot m\right)$$

Even when these expressions, with physical meaning, could fit the experimental data well, as shown in Figure 6b, they present an important drawback of not being invertible, since eq 9 leads to transcendental equations and eq 10, depending on the range of concentrations, is not an injective function. The inverse function is essential to express the concentration as a function of the measured conductance, principle on which the technique proposed here is based. Thus, a different fitting expression is proposed in this work, which, beyond the good fitting, is invertible, despite no physical significance could be stretched, even when a logarithmic relationship between electrical conductivity in solutions and electrolyte concentrations can be traced from the literature.^53,55,56^

9 $$G=A\;\ln\left(\left[rGO\right]/C_{0}-1\right)+B$$

where *G* is the conductance of the solution and *A*, *C*_0_, and *B* are the fitting parameters, whose values used for fitting experimental results are presented in Table 3.

**Table 3:** Fitting Parameters for the Calibration of Conductance

parameter	value	unit
*A*	(4.29509 ± 0.00002) × 10^–7^	S
*C*_0_	(2.000005 ± 0.000008) × 10^–7^	M
*B*	(5.348023 ± 0.000006) × 10^–6^	S

Figure 6b shows the different fitting curves (indicated in the figure) for the experimental conductance vs concentration curve. An excellent agreement between the experimental results and the proposed expression eq 11 is observed. Once the conductance of the solution is calibrated, the reduction reaction can be electrically monitored. Figure 6c shows the rGO concentrations computed from conductance measurements (blue symbols) and from UV–vis spectra (red symbols) and also the simulations using the proposed model (eqs 6–8). This model fits both sets of data accurately, since it describes the reaction beyond the technique used for its monitoring. Figure 6d shows a comparison between rGO concentrations obtained either from conductance measurements or by measuring the UV–vis spectrum of samples during reduction (blue squares). A straight line with a unit slope is also plotted for guiding the eye. This curve can be linearly fitted (*y* = *mx* + *b*), and the fitting parameters are shown in Table 4. A value of the slope close to 1 indicates good agreement between both techniques, even when the electrical monitoring seems to overestimate the rGO concentrations at the lowest and highest values, which might indicate that the calibration of conductance might be affected by a systematic error. Beyond this, the aim of this work, which is to analyze the viability of the technique, is achieved.

**Table 4:** Fitting Parameters for the Comparison between Techniques

parameter	value
*m*	(1.04 ± 0.03)
*b*	(−0.05 ± 0.04)

## Conclusions

In this work, a systematic study of the graphene oxide green reduction reaction was presented. Surprisingly, the reduction reaction kinetics seem to be scarcely addressed in the literature or, at least, some gaps persist. This is the case for the detailed modeling of the reaction kinetics for the reduction process. Furthermore, some aspects of the reduction process were apparently misinterpreted in earlier works when the reaction was monitored by optical means. For instance, instead of a redshift of the UV–vis peaks as the reaction proceeds, usually associated with structural changes, a diminishing characteristic of the GO and an increasing peak of the reduced GO are evident in this work. This observation is confirmed by the analysis of the deconvolution of the UV–vis spectra at the earlier stages of the reaction, which indicates the presence of GO and rGO before the latter becomes dominant. This fact is in good agreement with some results reported in the literature that mention the coexistence of GO and rGO during the reaction, as shown by AFM and XRD results. Moreover, our interpretation is more coherent with a picture of consumption of reactants and increasing concentration of the final product, whose concentrations are proportional to the UV–vis maximum absorbance. This process was modeled by considering a system of ordinary differential equations to take into account the main aspects of the kinetics, such as the fact that the reaction occurs while both reactants are present and ends when one of them is exhausted (nonstoichiometric reaction). The consumption/production rates were considered to be proportional to the product of the reactant concentrations. Since the UV–vis characteristics of the reactants are screened by the peak of the product, the model has successfully fitted the production of rGO. Finally, a novel technique for monitoring the reduction reaction based on measuring the electrical conductance is proposed and verified with success. The validation of the method was done by comparing the results obtained with the new technique with those obtained by the more traditional and accepted UV–vis technique. Nonetheless, the viability of this technique should be further explored for different values of the reactant concentrations, which might interfere with the electrical measurements.
